# Editorial: MicroRNAs and Muscle Cell Death in Cancer

**DOI:** 10.3389/fgene.2022.892136

**Published:** 2022-05-13

**Authors:** Jennifer M. Peterson, Francesco Chemello, Federica Calore

**Affiliations:** ^1^ School of Exercise and rehabilitation Sciences, University of Toledo, Toledo, OH, United States; ^2^ Department of Molecular Biology, University of Texas Southwestern Medical Center, Dallas, TX, United States; ^3^ Department of Cancer Biology and Genetics, The Ohio State University, Columbus, OH, United States

**Keywords:** microRNAs, muscle, muscle wasting (atrophy), cancer, extracellular vesicles, cachexia

MicroRNAs (miRNAs) are small non-coding RNA molecules that have gathered importance over the last decade as critical regulators of several biological processes, including differentiation, cell cycle regulation, aging and development (Fabbri et al., 2008). Most importantly, miRNAs are dysregulated in all types of cancers and are involved in the modulation of pivotal signaling pathways, hence contributing to tumor initiation and spread (Calin and Croce, 2006). Interestingly, several studies have also described miRNAs as regulators of the physiology of skeletal muscle cells as well as promoters of muscle cell death associated to atrophy in different types of pathological conditions: this Research Topic aims to collect and discuss most recent findings that can help to elucidate the role of miRNAs in such processes.

MiRNAs are known to regulate muscle development and function, however they also have been reported to modulate the onset of several muscle disorders (McCarthy et al., 2015; Goljanek-Whysall et al., 2012). On this regard, Singh et al. presented in their review detailed knowledge about the role of miRNAs in skeletal muscle myogenesis, myoblast differentiation and proliferation through their targeting of myogenic regulatory factors and signaling pathway modulation. Moreover, the Authors explored the role of miRNAs in different myopathies, focusing on their dysregulation in skeletal muscle cells during aging-dependent sarcopenia, their involvement as mediators of pathological pathways associated to skeletal muscle damage, fibrosis and regeneration in the Duchenne muscular dystrophy (DMD), as well as their role in cancer-associated cachexia.

Over the last decade, several studies have pointed out that miRNAs can be secreted by different types of cells into the extracellular space and then internalized by surrounding recipient cells, at the paracrine level, or reach even more distant sites thanks to the blood flow. Secreted miRNAs can indeed be isolated from different body fluids (Valadi et al., 2007; Smolarz and Widlak, 2021). For this reason, the so-called “circulating miRNAs” have recently gathered importance as potential, novel biomarkers. Rhabdomyosarcoma (RMS) is a common pediatric soft tissue sarcoma which depends on the inability of myogenic precursors to complete muscle differentiation (Leaphart and Rodeberg, 2007). Patient survival has been significantly improved thanks to combined therapeutic strategies, however some patients still experience relapse or present with metastatic rhabdomyosarcoma at diagnosis with poor prognosis, hence the urgent need for specific biomarkers. Blood-derived circulating miRNAs would perfectly fit to this purpose, as they could potentially provide crucial prognostic and/or diagnostic information in a non-invasive manner. For this purpose, Tombolan et al. compared by quantitative PCR the expression levels of 84 cancer-associated miRNAs (Cancer Panel) of plasma samples of RMS patients *versus* healthy donors.

Their findings revealed that low levels of miR-26a were significantly associated with a poorer outcome, from both the overall and progression-free survival standpoint, compared to patients presenting higher expression level of the miRNA in the blood, hence demonstrating the potential of circulating miR-26a as diagnostic and prognostic biomarker in children affected by this malignancy and providing important insights for a better understanding of such a terrible disease.

Cancer-associated cachexia is a very complex, multifactorial metabolic syndrome characterized by a dramatic, involuntary weight loss mainly attributed to skeletal muscle wasting, and is associated with several pathological conditions, including cancer (Fearon et al., 2011). Marceca et al. reviewed the role of miRNAs as post-transcriptional regulators in skeletal muscle physiology, discussing their role as mediators in muscle wasting associated to cancer cachexia. The Authors also reviewed both preclinical studies and miRNA profiling analyses performed on muscle biopsies isolated from cachectic cancer patients, pointing out dysregulated miRNAs as novel, potential biomarkers and therapeutic targets, and discussing the numerous discrepancies emerged among different studies, which reflect the complexity of the syndrome and the inconsistency of several xenograft models for cancer cachexia. Finally, the Authors explored the role of secreted, circulating miRNAs in cachexia, not only as potential prognostic and diagnostic tools, but also as critical modulators of apoptosis associated to muscle wasting.

Since the molecular mechanisms regulating cancer cachexia have not been fully understood yet, the use of novel technologies for mRNA transcriptome and miRNA profiling could unveil novel mechanisms and cellular networks involved in the initiation and progression of the syndrome. Moreover, by identifying multi-omics features within the same samples it would be possible to ascertain more accurate information. Fernandez et al. performed paired miRNA and mRNA co-profiling analyses on RNA samples isolated from the muscles of cachectic Lewis Lung Carcinoma-bearing mice and healthy control. Bioinformatic analyses (including motif, gene ontology enrichment, and interaction network analyses) allowed the identification of new extracellular matrix remodeling events modulated by miRNAs, hence contributing to muscle wasting associated with cachexia. Moreover, the Authors identified new transcription factors involved in cachexia, some of them implicated in cell cycle and myogenesis regulation, providing overall new hints for the development of novel, potential therapeutic strategies.

Collectively, articles in this research topic have introduced new insights and important perspectives about the role of miRNAs in muscle cell death associated with cancer, as well as other pathological conditions that we hope will inspire future, innovative investigations. We would like to thank all the Authors and Reviewers for their contribution to this research topic.
